# Predictors of mortality among elderly people living in a south Indian urban community; a 10/66 Dementia Research Group prospective population-based cohort study

**DOI:** 10.1186/1471-2458-10-366

**Published:** 2010-06-23

**Authors:** AT Jotheeswaran, Joseph D Williams, Martin J Prince

**Affiliations:** 1 Institute of Community Health, Voluntary Health Services, Chennai, India; 2 Centre for Global Mental Health, Health Service and Population Research Department, Institute of Psychiatry, P060, De Crespigny Park, London SE5 8AF, UK

## Abstract

**Background:**

Eighty percent of deaths occur in low and middle income countries (LMIC), where chronic diseases are the leading cause. Most of these deaths are of older people, but there is little information on the extent, pattern and predictors of their mortality. We studied these among people aged 65 years and over living in urban catchment areas in Chennai, south India.

**Methods:**

In a prospective population cohort study, 1005 participants were followed-up after three years. Baseline assessment included sociodemographic and socioeconomic characteristics, health behaviours, physical, mental and cognitive disorders, disability and subjective global health.

**Results:**

At follow-up, 257 (25.6%) were not traced. Baseline characteristics were similar to the 748 whose vital status was ascertained; 154 (20.6%) had died. The mortality rate was 92.5/1000 per annum for men and 51.0/1000 per annum for women. Adjusting for age and sex, mortality was associated with older age, male sex, having no friends, physical inactivity, smaller arm circumference, dementia, depression, poor self-rated health and disability. A parsimonious model included, in order of aetiologic force, male sex, smaller arm circumference, age, disability, and dementia. The total population attributable risk fraction was 0.90.

**Conclusion:**

A balanced approach to prevention of chronic disease deaths requires some attention to proximal risk factors in older people. Smoking and obesity seem much less relevant than in younger people. Undernutrition is preventable. While dementia makes the largest contribution to disability and dependency, comorbidity is the rule, and more attention should be given to the chronic care needs of those affected, and their carers.

## Background

Almost 80% of the world's deaths occur in low and middle income countries (LMIC), where estimation of cause of death is difficult, since most die at home without prior contact with health care professionals [1]. About 14.6 million deaths occurred in 2005 in the World Health Organisation's South-East Asia Region (SEARO) of which 6.8 million were among people aged 60 years and over [2]. Chronic diseases are now the leading cause of death in nearly all world regions, and older people contribute 72% of chronic disease deaths in LMIC [3]. Despite growing interest in chronic diseases in LMIC [4,5], there is limited information available on their prevalence and impact among older people.

Factors associated with mortality in late-life can be subsumed into five domains; demographic variables; socioeconomic status; social relationships; health behaviours and health status. There are few studies of predictors of mortality among older people in LMIC [6-8], and none that have comprehensively addressed all of the relevant domains. In Wuhan, China independent effects of older age, male gender, lower education and serious illness were noted, the protective effect of education being more pronounced among women [6]. Neither socioeconomic factors, nor social support was associated with mortality in the final adjusted models. In Matlab, Bangladesh, mortality was ascertained through regular population censuses between 1982 and 1996 [8]. Mortality rates were stable among older people in contrast to the secular decline in younger age groups. Those with better education and higher socioeconomic status experienced lower mortality, as did those living with children. In a cohort study of socioeconomically disadvantaged older people in and around Asan City in South Korea (a recently developed country), male gender and poor self-rated health were the main predictors of mortality [7]. Ischaemic heart disease and stroke predicted mortality in men only.

Risk factors for mortality may differ between older and younger people. In India, analysis of data from the Indian National Family Household Survey of 1998-99, including over half a million participants of all ages in 26 states, revealed a considerable attenuation of the effects of standard-of-living on mortality in people aged 65 years and older, for whom caste (an index of status rather than material resources) seemed to be more relevant [9]. Nearly 150,000 participants aged 35 years and over were recruited in a prospective cohort study in Mumbai, followed up for six years to assess the effects of tobacco use and body mass index on mortality [10]. Undernutrition was a stronger risk factor for mortality than obesity. The increased mortality associated with tobacco use was mainly confined to smokers rather than those using smokeless products. Another report from the same study indicated that the relative risk for mortality associated with smoking cigarettes or bidi was substantially smaller in those aged 50 and over compared with younger participants [11]. In developed countries the association between mortality and lower socioeconomic status is well documented [12], including among older people [13,14]. However, some studies suggest that the socioeconomic gradient may be attenuated among older age groups [15]. Among older people evidence suggests increased mortality among those who smoke, have a poor quality diet, and are physically inactive [16]. In contrast to younger age groups, undernutrition [17,18] is more consistently associated with mortality than obesity [19]. Depression and dementia have been extensively studied and are prominent independent predictors of mortality among older people. Meta-analyses suggest relative risks of 1.81 (95% CI 1.58-2.07) for depression [20], and 2.63 (95% CI 2.17-3.21) for dementia [21]. The effect of dementia seems to attenuate in the oldest old [22]. The effect sizes seem to be larger in LMIC; 5.16 in Brazil [23] and 2.83 in Nigeria [24]. There is an extensive literature attesting to the independent effects of lack of social relationships upon mortality among older people [25,26], with effect sizes comparable to health risk factors such as high blood pressure, smoking and obesity [27]. Functional and perceived aspects of social support may be even more salient to mortality risk [28]. Subjective well being and self-rated health were inversely associated with mortality among older people in several studies [29-31], although there are also negative reports [32,33].

In summary, the large majority of deaths worldwide occur in LMIC, chronic diseases are now the leading cause of death in these regions, and most deaths from chronic diseases in LMIC are deaths of older people. Findings from studies carried out in developed countries suggest that predictors of mortality identified in younger age groups may not generalise to older people, and, of course, associations observed among older people in more developed settings may not generalise to LMIC. Identification of modifiable factors more proximally associated with mortality in late-life may lead to improvements in the quality as well as the quantity of life of older people. In this paper we assess the relative importance of sociodemographic characteristics, socioeconomic factors, health risk behaviours, physical, mental and cognitive morbidities, and the impact of these chronic conditions on health-related quality of life as predictors of mortality among people aged 65 years and over living in urban catchment areas in Chennai, south India.

## Methods

### Settings and study design

The 10/66 Dementia Research Group's (10/66 DRG) population-based studies of ageing and dementia in LMIC included an urban Indian site comprising five geographically defined catchment areas, Kandhanchavadi, Perungudi, Thoraipakkam, Palavakkam, and Kottivakkam in south Chennai. The design of the baseline and follow-up phases of the 10/66 DRG research program have been described in detail elsewhere [34]. The baseline survey comprised a comprehensive one phase assessment of all residents aged 65 years and over living in the defined catchment areas, identified by systematic door-knocking. In Chennai, 1005 participants were recruited into the baseline survey carried out between 2004 and 2006, representing 72% of all those eligible to participate. We carried out a follow-up of all baseline participants between December 2007 and July 2008, seeking to determine their vital status, and for those that had died, to determine their date of death and complete verbal autopsy interviews with relatives. Tracing was facilitated by the collection, at baseline of names and addresses of friends and family living in other households nearby. A minimum of three visits were made before a participant was declared to be untraceable. The baseline and follow-up studies were approved by King's College Research Ethics Committee and the Research Ethics Committee at Voluntary Health Services, Chennai, India, and participation was on the basis of informed signed consent.

### Measures

Full details of the assessments used in the baseline surveys are provided in another open access publication [34]. Here we describe only those assessments relevant to the analyses presented in this paper.

#### Sociodemographic status

Participants' ages were established during the baseline interview, from stated age, official documentation, informant report, and, in the case of discrepancy, age according to an event calendar. We also recorded the participant's gender, marital status and level of education. Socioeconomic position was ascertained according to the number of household assets (cars; television; fridge and/or freezer; mains water; mains electricity; telephone; plumbed toilet; plumbed bathroom), declared income, and the presence or absence of food insecurity (sometimes or often having gone without food in the last month).

#### Health status

1) Diagnoses ascertained by clinical interview or examination

a) Dementia according to either our 10/66 dementia diagnosis algorithm (developed, calibrated and pre-validated cross-culturally in our 26 centre LMIC pilot study [35]) or Diagnostic and Statistical Manual, 4^th ^edition (DSM-IV) dementia criterion applied using an operationalised computer algorithm [36]. We assessed the severity of dementia (questionable, mild, moderate or severe) using the Clinical Dementia Rating Scale (CDR) [37]. Amnestic mild cognitive impairment (MCI) was defined as objective and subjective memory impairment, with no impairment in activities of daily living and not meeting the criteria for dementia [38].

b) Depression - ICD-10 depressive episode (mild, moderate or severe), ascertained using the Geriatric Mental State [39]. Those scoring four or more on the EURO-D depression scale [40], but not having symptoms meeting the criteria for ICD-10 depression were defined as having 'sub-syndromal depression'.

c) Hypertension - physical examination was performed to record systolic and diastolic resting blood pressure (average of two). Hypertension was defined as having been told by a doctor that you have hypertension and/or meeting European Society of Hypertension criteria (systolic blood pressure > = 140 mm Hg and/or diastolic blood pressure > = 95 mm Hg).

d) Chronic obstructive pulmonary disease, defined as having a chronic cough, productive of sputum for three or more months

2) Self-reported diagnoses - stroke, angina and myocardial infarction ("have you ever been told by a doctor that you had a stroke/heart attack/angina/diabetes?")

3) Self-reported physical impairments - arthritis or rheumatism; eyesight problems; hearing difficulty or deafness; persistent cough; breathlessness, difficulty breathing or asthma; high blood pressure; heart trouble or angina; stomach or intestine problems; faints or blackouts; paralysis, weakness or loss of one leg or arm; skin disorders [41]. Each impairment was rated as present if it interfered with activities 'a little' or 'a lot'.

4) Disability - activity limitation and participation restriction measured by the WHODAS 2.0 [42], developed by the World Health Organization as a culture-fair assessment tool for use in cross-cultural comparative epidemiological and health services research.

5) Subjective global health rated on a five point scale - very good/good/moderate/bad/very bad

#### Lifestyle risk factors

a) Alcohol use - patterns of drinking behaviour were ascertained from self-reports of units of alcohol consumed per week currently and before the age of 65 years. These data were used to identify three groups; long-term abstainers, previous drinkers who abstain after the age of 65, and consistent drinkers. Those who answered yes to either or both of the questions 'Has there ever been a period of several years when you would have said that you were a heavy drinker?' and 'Have you ever had treatment or help for drinking from a doctor or some other agency?' were identified as heavy problem drinkers.

b) Tobacco use - we enquired after smoking behaviour (coded in this analysis as never, ex- and current smokers) and use of smokeless tobacco

c) Nutrition - reported current consumption of meat and fish; mid-upper arm circumference and waist circumference in centimetres

d) Underactivity - taking no walks of 500 metres or more in the past month

### Analyses

All data was collected and entered into EPIDATA software and data analysis was performed using STATA version 10. We calculated crude mortality rates per 1000/year for each age group and both genders. India urban national mortality (1999) [43] and US national mortality rates (2005) [44] were used to calculate age and sex standardized mortality ratios (SMR) to compare the mortality experience of our cohort with that of these populations. Chi^2 ^tests or t-tests were performed as appropriate to assess the statistical significance of differences in baseline characteristics between those participants whose vital status at follow-up was ascertained or unknown. The characteristics of those followed up successfully were then described by gender and by household assets. For the bivariate analyses of associations with mortality we report the cumulative mortality by exposure level for sociodemographic variables, lifestyle and other chronic disease risk factors, and health status (diagnoses, impairments and health related quality of life) with crude hazard ratios derived from Cox proportional hazard regression and logrank tests for statistical significance. We also report each of the hazard ratios adjusted for age and sex. Next we derived a parsimonious model for the prediction of mortality in the sample using forwards and backwards stepwise hazard regression procedures based on likelihood ratio testing at each step, initially entering all variables found to be significantly associated with mortality in either the crude or age and sex adjusted analyses. We then fitted the same model using Poisson regression to estimate the population attributable risk fraction (PARF) for each of the exposures in the parsimonious model, using the Stata aflogit command, which estimates the PARF from within a logistic regression framework, enabling confounding to be taken into account, and providing a robust estimation of the summary (combined) PARF for the full set of exposures in the model.

## Results

### Sample characteristics

We were able to determine the status of 748 (74.4%) of the 1005 baseline participants, 594 (74.4%) of whom were alive and 154 (25.6%) had died. The remaining 257 could not be traced. This loss to follow-up was mainly accounted for by the construction of an information technology superhighway through the catchment area. There were no large or statistically significant differences in the sociodemographic, socioeconomic or health circumstances at baseline of those whose vital status was and was not ascertained at follow-up (Table 1), other than that those not followed-up had marginally smaller waist circumferences (p = 0.05). For those who were followed up, men were older than women, had more education, were more likely to be currently married, had more household assets, were more likely to smoke, to drink and to engage in problem drinking, and to report ischaemic heart disease (Table 1). However, women had worse self-reported health and higher disability scores. Also among those followed up, those with more household assets were older, and more likely to be male, better educated, and currently married. They were less likely to smoke. However, they had larger waists and upper arm circumferences, were more likely to be hypertensive, and to report diabetes, and three or more limiting chronic illnesses (Table 1).

**Table 1:** Baseline sample characteristics by ascertainment of vital status at follow up, and by gender and household assets for those whose vital status was known.

	Baseline sample characteristics by ascertainment of vital status at follow up			**Baseline sample characteristics by gender, for those whose vital status was known**.			**Baseline sample characteristics by household assets, for those whose vital status was known**.		
	**Vital Status known** **(n = 748)**	**Vital Status not known** **(n = 257)**	**χ^2^, df (p-value)**	**Female** **(n = 422)**	**Male** **(n = 316)**	**χ^2^, df (p-value)**	**Fewest assets - 4 or fewer** **(n = 459)**	**Most assets - 5 or more** **(n = 289)**	**χ^2^, df (p-value)**

Age - mean (SD)	71.4 (6.1)	71.3 (6.2)	0.3^1^, 1001 (0.78)	71.0 (5.8)	71.8 (6.3)	1.9^1^, 644 (0.05)	70.9 (5.9)	72.2 (6.2)	2.9^1^, 744 (0.004)

Female (%)	422 (57.2%)	149 (59.1%)	0.3, 1 (0.59)	-	-	-	272 (60.0%)	150 (52.6%)	3.9, 1, (0.05)

**Education**			0.0, 1 (0.98)			71.9, 1 (< 0.001)			101.5, 1, (< 0.001)

None	314 (42.1%)	114 (44.4%)		240 (57.0%)	72 (22.8%)		245 (53.5	69 (24.0%)	

Some	178 (23.9%)	56 (21.8%)		78 (18.5%)	98 (31.0%)		111 (24.3%)	67 (23.3%)	

Primary	160 (21.4%)	52 (20.2%)		74 (17.6%)	84 (26.6%)		77 (16.8%)	83 (28.8%)	

Secondary	66 (8.8%)	21 (8.2%)		20 (4.8%)	44 (13.9%)		23 (5.0%)	43 (14.9%)	

Tertiary	28 (3.8%)	14 (5.4%)		9 (2.1%)	18 (5.7%)		2 (0.4%)	26 (9.0%)	

**Marital status**			6.0, 3 (0.11)			184.0, 3 (< 0.001)			10.1, 3, (0.02)

Never married	12 (1.6%)	9 (3.5%)		5 (1.2%)	6 (1.9%)		8 (1.7%)	4 (1.4%)	

Married	382 (51.1%)	141 (55.3%)		125 (29.6%)	250 (79.1%)		215 (46.8%)	167 (58.0%)	

Widowed	330 (44.2%)	96 (37.6%)		275 (65.2%)	54 (17.1%)		218 (47.5%)	112 (38.9%)	

Divorced/separated	23 (3.1%)	9 (3.5%)		17 (4.0%)	6 (1.9%)		18 (3.9%)	5 (1.7%)	

**Assets**			0.86, 1 (0.35)			5.0, 1 (0.03)			

1^st ^quarter	90 (12.0%)	42 (16.5%)		52 (12.4%)	37 (11.7%)		-	-	

2^nd ^quarter	368 (49.3%)	122 (48.0%)		219 (52.0%)	144 (45.6%)		-	-	

3^rd ^quarter	105 (14.1%)	25 (9.8%)		63 (15.0%)	42 (13.3%)		-	-	

4^th ^quarter	184 (24.6%)	65 (25.6%)		67 (20.7%)	93 (29.4%)		-	-	

**Smoking **:			0.9, 2 (0.65)			161.5, 2 (< 0.001)			19.2, 2, (< 0.001)

Never smoker	548 (73.8%)	182 (71.7%)		381 (90.9%)	162 (51.3%)		316 (69.1%)	232 (81.1%)	

Ex-smoker	65 (8.7%)	21 (8.3%)		0 (0.0%)	62 (19.6%)		39 (8.5%)	26 (9.1%)	

Current smoker	130 (17.5%)	51 (20.1%)		38 (9.1%)	92 (29.1%)		102 (22.3%)	28 (9.8%)	

**Alcohol use**			0.4, 1 (0.51)			102.1, 1 (< 0.001)			0.6, 1, (0.43)

Always abstinent	559 (83.9%)	174 (83.7%)		364 (99.2%)	194 (65.1%)		338 (83.0%)	221 (85.3%)	

Moderate drinker, abstinent after the age of 65	45 (6.8%)	15 (7.2%)		2 (0.5%)	43 (14.4%)		29 (7.1%)	16 (6.2%)	

Consistent moderate drinker	26 (3.9%)	13 (6.3%)		0 (0.0%)	26 (8.7%)		16 (3.9%)	10 (3.9%)	

Heavy drinker and/or sought treatment	36 (5.4%)	6 (2.9%)		1 (0.3%)	35 (11.7%)		24 (5.9%)	12 (4.6%)	

Physically inactive	160 (21.5%)	58 (23.0%)	0.3, 1 (0.61)	101 (23.9%)	58 (18.4%)	3.3, 1 (0.07)	107 (23.4%)	53 (18.5%)	2.5, 1, (0.11)

Waist circumference - mean (SD)	83.2 (11.1)	81.5 (11.5)	2.0^1^, 987 (0.05)	82.5 (10.9)	84.0 (11.1)	1.7^1^, 725 (0.08)	81.4 (10.9)	85.8 (11.2)	5.2^1^, 744, (< 0.001)

Arm circumference - mean (SD)	23.8 (4.2)	23.3 (3.8)	1.6^1^, 991 (0.11)	23.4 (3.9)	24.2 (3.9)	2.8^1^, 728 (0.006)	22.8 (3.4)	25.3 (4.8)	8.4^1^, 744, (< 0.001)

ICD-10 Depression	25 (3.3%)	14 (5.4%)	2.3, 1 (0.13)	13 (3.1%)	12 (3.8%)	0.3, 1 (0.59)	18 (3.9%)	7 (2.4%)	1.2, 1, (0.27)

Dementia	54 (7.2%)	21 (8.2%)	0.3, 1 (0.62)	33 (7.8%)	21 (6.6%)	0.4, 1 (0.54)	37 (8.1%)	17 (5.9%)	1.3, 1, (0.26)

Hypertension	454 (60.7%)	144 (56.0%)	1.7, 1 (0.19)	261 (61.8%)	188 (59.5%)	0.4, 1 (0.52)	257 (56.0%)	197 (68.2%)	11.0, 1, (0.001)

Stroke	17 (2.3%)	3 (1.2%)	1.2, 1 (0.27)	6 (1.4%)	9 (2.8%)	1.8, 1 (0.17)	9 (2.0%)	8 (2.8%)	0.5, 1, (0.47)

Ischaemic heart disease	38 (5.1%)	11 (4.3%)	0.3, 1 (0.61)	14 (3.3%)	24 (7.6%)	6.8, 1 (0.009)	19 (4.1%)	19 (6.6%)	2.2, 1, (0.14)

Diabetes	92 (12.3%)	29 (11.3%)	0.2, 1 (0.66)	51 (12.1%)	40 (12.7%)	0.1, 1 (0.81)	46 (10.0%)	46 (16.0%)	5.8, 1, (0.02)

Three or more limiting physical impairments	30 (4.0%)	11 (4.3%)	0.0, 1 (0.85)	16 (3.8%)	13 (4.1%)	0.1, 1 (0.82)	10 (2.2%)	20 (6.9%)	10.4, 1, (0.001)

**Self-rated health**			2.4, 1 (0.12)			14.3, 1 (< 0.001)			0.0, 1, 0.95

Very good	321 (43.0%)	97 (37.9%)		165 (39.1%)	153 (48.4%)		197 (42.9%)	124 (43.1%)	

Good	269 (36.0%)	98 (38.3%)		149 (35.3%)	115 (36.4%)		165 (35.9%)	104 (36.1%)	

Moderate	126 (16.9%)	46 (18.0%)		83 (19.7%)	43 (13.6%)		78 (17.0%)	48 (16.7%)	

Bad or very bad	31 (4.1%)	15 (5.9%)		25 (5.9%)	5 (1.6%)		19 (4.1%)	12 (4.2%)	

Disability - mean (SD)	10.7 (15.2)	10.1 (16.2)	0.5^1^, 999 (0.61)	12.1 (16.1)	8.6 (13.1)		10.9 (15.7)	10.4 (14.3)	0.4^1^, 744, (0.70)

1. F-value from t-test

### Mortality rate

One hundred and fifty four deaths were recorded. For 34 of these the informant did not know the place of death, 102 (85.0%) occurred at home and 18 (15.0%) in hospital. For 83 (76.9%) of the 108 deaths for which such information was available, no treatment of any kind was said to have been provided for the final illness. The overall mortality rate was 70.0/1000 per annum, 92.5/1000 pa for men and 51.0/1000 pa for women. The mortality rate increased steadily with age for both sexes (Table 2). After indirect standardization for age and gender, the observed mortality in our cohort was similar to that for urban India for women (SMR 103, 95% CI 72 to 146) but higher than expected for men (SMR 158, 95% CI 112 to 229). The observed mortality was higher than that for the USA among both women (SMR 198, 95% CI 132 to 315) and men (SMR 231, 95% CI 159 to 359).

**Table 2:** Crude mortality rates, per 1000 person years, by age and sex

Sex (10 MV^1^)	Women			Men			Both sexes combined		
**Age** **(3 MV^1^)**	**Deaths**	**Person years**	**Mortality rate (per 1000 person years)**	**Deaths**	**Person years**	**Mortality rate (per 1000 person years)**	**Deaths**	**Person years**	**Mortality rate (per 1000 person years)**

65-69	19	561.1	33.9	21	388.6	54.0	42	960.7	43.7

70-74	21	421.5	49.8	22	247.5	88.9	44	680.1	64.7

75-79	11	148.6	74.0	19	163.7	116.1	30	312.3	96.1

80+	13	137.0	94.9	21	94.8	221.5	37	238.2	155.3

All ages combined	65	1274.4	51.0	83	897.3	92.5	154	2200.2	70.0

### Bivariate analysis - baseline predictors of mortality

Older age and male sex were associated with increased mortality with little mutual confounding (Table 3). Neither educational status, income, receipt of a pension, household assets, nor food insecurity predicted mortality. Marital status was not associated with mortality; however, there was some evidence that lacking friends or contact with friends was associated with an increased mortality risk.

**Table 3:** Mortality by baseline sociodemographic characteristics:

Socio demographic characteristic	Cumulative mortality	Hazard ratio (95%CI)	Hazard ratio (95% CI) Adjusted for Age and sex	Log-rank test
**Age group (MV^1 ^= 2)**				χ^2 ^= 38.3, df(3), p = <0.001

65-69	42/268 (13.5%)	Ref	Ref	

70-74	44/231 (19.0%)	1.5 (0.9-2.2)	1.5 (1.0-2.3)	

75-79	30/112 (26.8%)	2.3 (1.4-3.7)	2.2(1.4-3.6)	

80+	37/92 (40.2%)	3.5 (2.2-5.5)	3.5 (2.2-5.5)	

**Sex (MV = 10)**				χ^2 ^= 13.9, df (1), p = 0.0002

Female	65/422 (15.4%)	Ref	Ref	

Male	83/316 (26.3%)	1.8 (1.3-2.5)	1.8 (1.3-2.5)	

**Marital status (MV = 1)**				χ^2 ^= 1.73, df (3),p = 0.63

Never married	4/12(33.3%)	Ref	Ref	

Married	87/382 (22.7%)	0.5 (0.2-1.6)	0.5 (0.1-1.4)	

Widowed	59/330(17.8%)	0.5 (0.2-1.5)	0.6 (0.2-1.8)	

Divorced/separated	4/23(17.4%)	0.4 (0.1-1.7)	0.4 (0.1-1.8)	

**Living arrangements (MV = 0)**				χ^2 ^= 0.2, df (1), p = 0.66

Alone or with spouse only	22/117 (18.8%)	0.9 (0.5-1.4)	0.9 (0.5-1.4)	

With others	132/631 (20.9%)	Ref	Ref	

**Educational level (MV = 2)**				χ^2 ^= 0.0, df (1),p = 0.99

None	68/314 (21.6%)	Ref	Ref	

Some	33/178 (18.5%)	0.9 (0.6-1.4)	0.7(0.5-1.2)	

Primary	35/160 (21.8%)	1.0 (0.7-1.6)	0.8 (0.5-1.2)	

Secondary & Higher	17/94(18.1%)	0.9 (0.5-1.6)	0.6 (0.3-1.1)	

**Have friends (MV = 2)**				χ^2 ^= 4.70, df(1), p = 0.03

Yes	66/382 (17.2%)	Ref	Ref	

No	87/364 (24%)	1.4 (1.0-1.9)	1.3 (0.9-1.8)	

**Frequency of meeting friends (MV = 2)**				χ^2 ^= 4.2, df(4), p = 0.3

Daily	51/288 (17.7%)	Ref	Ref	

2-3 times/week	7/42 (16.6%)	1.0 (0.4-2.3)	1.1 (0.5-2.5)	

At least weekly	5/31 (16.1%)	1.1 (0.4-2.8)	1.3 (0.5-3.3)	

Less often	3/16 (18.7%)	1.2 (0.3 -3.7)	1.1 (0.3-3.6)	

No friends/never see	87/369 (23.6%)	1.4 (1.0 -2.0)	1.5 (1.0-2.1)	

**Socioeconomic status**				

**Declared Income (MV = 0)**				χ^2 ^= 2.65, df(2), p = 0.26

< Rs.3000	104/446 (23.3%)	Ref	Ref	

Rs.3000 to 6000	28/197 (14.2%)	0.9 (0.6 -1.4)	1.0 (0.6-1.5)	

> Rs. 6000	22/105 (20.9%)	1.4 (0.8 -2.2)	1.3 (0.8-2.2)	

**Number of Assets (MV = 1)**				χ^2 ^= 0.75, df(1), p = 0.39

1^st ^quarter (fewest)	16/90 (17.7%)	Ref	Ref	

2^nd ^quarters	79/368 (21.4%)	1.4 (0.8 -2.4)	1.4 (0.8 -2.5)	

3^rd ^quarters	19/105 (18.1%)	1.4 (0.7-2.7)	1.3 (0.6 -2.5)	

4^th ^quarter (highest)	39/184 (21.2%)	1.5 (0.8 -2.7)	1.3 (0.7 -2.3)	

**Pension receipt (MV = 0)**				χ^2 ^= 0.00, df(1), p = 0.96

Yes	135/653 (20.6%)	Ref	Ref	

No	19/95 (20.0%)	1.0 (0.6-1.6)	0.9 (0.5 -1.4)	

**Food insecurity (MV = 3)**				χ^2 ^= 0.28, df(1), p = 0.59

No	121/598 (20.2%)	Ref	Ref	

Yes	33/147 (22.4%)	1.1 (0.7 -1.6)	1.1 (0.7 -1.6)	

1. MV = Number of missing values

There was an elevated mortality risk among ever smokers compared with those using smokeless tobacco and non-smokers, and a significant trend for an increasing risk with increasing levels of drinking from total abstinence through to heavy problem drinkers (Table 4). However, each of these associations attenuated, and was no longer statistically significant after controlling for age and sex. Physical inactivity conferred an increased risk. While dietary intake was not associated with mortality, there was a non-linear association between waist circumference and mortality with the increased risk confined to those in the second quarter of the population distribution, and a strong linear trend for increased risk associated with smaller mid upper arm circumferences.

**Table 4:** Mortality by lifestyle and other chronic disease risk factors

Life style factors	Cumulative mortality	Hazard ratio (95%CI)	Hazard ratio (95% CI) Adjusted for Age + Gender	Log-rank test
				

**Smoking (MV^1 ^= 5)**				χ^2 ^= 7.04, df(2),p = 0.02

Never smoked	102/548 (18.6%)	Ref	Ref	

Smokeless tobacco	7/36 (19.4%)	0.9 (0.4 -2.0)	0.7 (0.3 -1.7)	

Smoking tobacco	43/158 (27.1%)	1.6 (1.1 -2.2)	1.1 (0.7 -1.6)	

**Alcohol use (MV = 82)**				χ^2 ^= 5.65, df(1),p = 0.02

Always abstinent	108/559 (19.3%)	Ref	Ref	

Moderate drinker, abstinent after the age of 65	8/45 (17.9%)	0.8 (0.4-1.7)	0.6 (0.3-1.2)	

Consistent moderate drinker	7/26 (26.9%)	1.4 (0.7-3.1)	1.0 (0.4-2.1)	

Heavy drinker and/or sought treatment for alcohol problem	14/36 (38.9%)	2.1 (1.2-3.7)	1.4 (0.8-2.6)	

**Diet and nutrition**				

**Fish in diet (MV = 3)**				χ^2 ^= 0.77, df (2),p = 0.68

Never	26/139 (18.7%)	Ref	Ref	

Some days	103/503 (20.4%)	1.0 (0.7 -1.6)	1.1 (0.7 -1.7)	

Most of the days	25/103 (24.2%)	1.2 (0.7 -2.2)	1.3 (0.7 -2.3)	

**Meat in diet (MV = 2)**				χ^2 ^= 0.07, df(2),p = 0.96

Never	34/180 (18.8%)	Ref		

Some days	100/474 (21.1%)	1.0 (0.7 -1.5)	1.0 (0.7 -1.5)	

Most of the days	20/92 (21.7%)	1.0(0.5 -1.7)	1.0 (0.6 -1.8)	

**Physical activity (MV = 3)**				χ^2 ^= 7.32, df (1),p = 0.006

Yes	124/637 (19.4%)	Ref	Ref	

No	28/107 (28.0%)	1.7 (1.1 -2.5)	1.5 (1.0 -2.3)	

**Arm circumference (MV = 9)**				χ^2 ^= 10.1, df(1),p = 0.001

1^st ^quarter	59/200 (29.5%)	Ref	Ref	

2^nd ^quarter	38/185 (20.5%)	0.6 (0.4 -1.0)	0.6 (0.4-0.9)	

3^rd ^quarter	25/156 (16.0%)	0.5 (0.3 -0.9)	0.5 (0.3-0.9)	

4^th ^quarter	30/198 (15.1%)	0.5 (0.3 -0.7)	0.4 (0.3-0.7)	

Per centimetre	-	0.90 (0.85-0.94)	0.89 (0.84-0.94)	

**Waist circumference (MV = 12)**				χ^2 ^= 14.6, df(3),p = 0.002

1^st ^quarter	33/174 (19.0%)	(Ref)	(Ref)	

2^nd ^quarter	58/189 (30.7%)	1.8 (1.2-2.8)	1.7 (1.1-2.7)	

3^rd ^quarter	31/187 (16.6%)	0.9 (0.5-1.5)	0.9 (0.5-1.5)	

4^th ^quarter	29/186 (15.6%)	0.9 (0.5-1.4)	0.9 (0.5-1.4)	

1. MV = Number of missing values

Neither hypertension, ischaemic heart disease, stroke, diabetes, chronic obstructive pulmonary disease, or the number of limiting physical impairments was associated with mortality, either in the bivariate analysis, or after controlling for age and sex (Table 5). Indeed the only diagnoses or impairments associated with mortality were depression, and cognitive impairment and dementia, in both cases with increasing mortality risk with increasing levels of morbidity, independent of the effects of age and sex.

**Table 5:** Mortality by baseline health status - diagnoses and impairments

Morbidities & Life style	Cumulative mortality	Hazard ratio (95%CI)	Hazard ratio (95% CI) Adjusted for Age + Gender	Log-rank test
***Survey clinical diagnosis***				

**Dementia (MV^1 ^= 0)**				χ^2 ^= 25.0, df(1) p = < 0.001

No	129/694 (18.6%)	Ref	Ref	

Yes	25/54 (46.3%)	2.8 (1.8 -4.3)	2.3 (1.5 -3.7)	

**Cognitive Impairment (MV = 0)**				χ^2 ^= 37.4, df(1),p = <0.001

Cognitively healthy	122/672 (18.2%)	Ref	Ref	

Mild Cognitive Impairment	7/26 (26.9%)	1.9 (0.9-4.0)	1.9 (0.9 -4.2)	

CDR questionable dementia	15/34 (44.1%)	2.8 (1.6-4.8)	2.1 (1.2 -3.7)	

CDR mild moderate & severe dementia	10/16 (62.5%)	5.1 (2.7-9.7)	4.1 (2.1 -7.9)	

**Depression (MV = 0)**				χ^2 ^= 9.2, df(1), p = 0.002

No depression	81/446 (18.2%)	Ref	Ref	

Depression subcase	65/277 (23.5%)	1.5 (1.1-2.2)	1.6 (1.2-2.3)	

ICD 10 depression case	8/25 (32.0%)	2.2 (1.1 -4.5)	2.1 (1.0 -4.3)	

**Hypertension (MV = 0)**				χ^2 ^= 0.8, df(1),p = 0.59

No	69/294 (23.4%)	Ref	Ref	

Yes	85/454 (18.7%)	0.8 (0.6 -1.1)	0.9 (0.6 -1.3)	

**Chronic Obstructive Pulmonary Disease**				χ^2 ^= 0.20, df(1),p = 0.65

No	151/734 (20.5%)	Ref	Ref	

Yes	3/13 (23.1%)	1.3 (0.4-4.0)	1.6 (0.5 -5.2)	

***Self-reported physician diagnosis***				

**Stroke (MV = 1)**				χ^2 ^= 1.87, df(1),p = 0.17

No	149/731 (20.3%)	Ref	Ref	

Yes	5/16 (31.2%)	1.8 (0.7-4.5)	1.6 (0.6 -4.1)	

**Diabetes (MV = 1)**				χ^2 ^= 0.57, df(1), p = 0.44

No	138/655 (21.0%)	Ref	Ref	

Yes	16/92 (17.4%)	0.8 (0.4 -1.3)	0.7 (0.4 -1.3)	

**Ischaemic heart disease (MV = 1)**				χ^2 ^= 0.08, df(1),p = 0.78

No	147/709 (20.7%)	Ref	Ref	

Yes	7/38 (18.4%)	0.8(0.4 -1.9)	0.9 (0.4 -1.9)	

**Number of self-reported limiting physical impairments (MV = 1)**				χ^2 ^= 3.65, df(2),p = 0.16

None	89/479 (18.5%)	Ref	Ref	

One or two	58/238 (24.3%)	1.3(0.9-1.9)	1.4 (1.0 -1.9)	

More than three	7/30 (23.3%)	1.3(0.6-2.8)	1.1(0.4 -2.6)	

**Time taken to walk 10 metres (per second) (MV = 6)**	-	1.02 (1.01-1.03)	1.02 (1.01-1.04)	χ^2 ^= 5.5, df(1) p = 0.02

**Subjective global health (MV = 1)**				χ^2 ^= 11.0, df(3),p = 0.01

Very healthy	63/321 (19.6%)	Ref	Ref	

Good	51/269(18.9%)	0.9(0.6 -1.3)	0.9(0.6-1.3)	

Moderate	27/126(21.4%)	1.0(0.6 -1.6)	1.0(0.7 -1.7)	

Not very healthy	13/31 (41.9%)	2.5(1.3-4.5)	2.9 (1.6 -5.4)	

WHODAS 12 disability score (per unit)	-	1.023 (1.015-1.030)	1.024 (1.015-1.032)	χ^2 ^= 37.3, df(1) p < 0.001

1. MV = Number of missing values

### A parsimonious model to predict mortality

The final parsimonious Cox proportional hazards model included the effects of gender, age, dementia/cognitive impairment, and mid upper arm circumference (Model 1 - Table 6). According to likelihood ratio tests, no other variables could be added to the model with significant improvement (with the exception of WHODAS disability score) or removed from the model without significant deterioration in model fit. Inclusion of WHODAS disability score (Model 2) further improved the fit of the model, but with some attenuation of the effect of cognitive impairment/dementia. Both models met proportional hazards assumptions, both with respect to the global test (Table 6) and individual covariates. In terms of population attributable risk fractions the predictors of mortality with the strongest aetiologic force were, in descending order, male sex, mid upper arm circumference, age, disability, and cognitive impairment/dementia. Both models were highly predictive of future mortality; for model 2, including the effects of disability, the total adjusted PARF was 0.90 (95% CI 0.79-0.95)

**Table 6:** Cox Proportional Hazard predictors of mortality among older adult

	Model 1		Model 2	
**Risk exposure**	**Hazard Ratio (95% CI) adjusted for all variables in the model** **(n = 727)**	**Adjusted Population Attributable Risk Fraction** **(95% CI)**	**Hazard Ratio (95% CI) adjusted for all variables in the model** **(n = 726)**	**Adjusted Population Attributable Risk Fraction** **(95% CI)**

**Male sex**	2.0 (1.4-2.8)	0.55 (0.30-0.71)	2.1 (1.5-3.0)	0.59 (0.36-0.74)

**Arm circumference**	0.90 (0.85-0.95)	0.43 (0.19-0.60)	0.90 (0.86-0.95)	0.44 (0.21-0.60)

Age		0.45 (0.27-0.59)		0.41 (0.21-0.56)

65-69	1 (ref)		1 (ref)	

70-74	1.4 (0.9-2.1)		1.4 (0.9-3.6)	

75-79	1.9 (1.2-3.1)		1.7 (1.1-2.8)	

80+	2.7 (1.7-1.7)		2.5 (1.5-4.0)	

**Cognitive Impairment/Dementia**		0.10 (0.06-0.13)		0.07 (0.04-0.10)

Cognitively healthy	1 (ref)		1 (ref)	

Mild Cognitive Impairment	1.4 (0.9-2.1)		1.7 (0.8-3.6)	

CDR questionable dementia	1.9 (1.2-3.1)		1.5 (0.8-2.7)	

CDR mild dementia	3.2 (1.6-6.4)		2.4 (1.2-4.8)	

**WHODAS disability score**	Not included	Not included	1.02 (1.01-1.03)	0.21 (0.14-0.28)

Total Population Attributable risk fraction	-	0.87 (0.74-0.93)	-	0.90 (0.79-0.95)

Global test of proportional hazards assumption	Chi squared = 6.1,9 df, p = 0.73		Chi squared = 7.1, 10 df, p = 0.72	

### Mortality and gender

Given the strong observed associations in our sample between sex and sociodemographic factors (age, education, assets, marital status) health related factors (smoking, alcohol use, ischaemic heart disease) and health related quality of life (subjective global health rating and disability), we decided to assess the extent to which increased mortality observed among men (crude hazard ratio 1.82, 95% CI, 1.32-2.52) might be mediated through any of these variables. The effect of male gender did not attenuate after adjusting for age (1.80), education (2.04), assets (1.84), marital status (2.04), nor for all of these sociodemographic variables simultaneously (1.90). Neither did the effect attenuate after controlling for smoking (1.80) or ischaemic heart disease (1.84); some attenuation was seen after adjusting for alcohol use (1.57), and for all of these health variables simultaneously (1.60). Neither subjective global health rating (1.98), disability (2.11) nor both variables together (2.15) attenuated the effect of male sex. The effect of male sex in the final fully adjusted model, including all of the above mentioned sociodemographic, health and health related quality of life variables was 2.05 (95% CI, 1.37-3.32).

## Discussion

The strengths of this study are; first the focus upon older people, among whom, in India and other LMIC there are few data on the extent, pattern and predictors of mortality; and second the relatively comprehensive coverage of potentially relevant exposures at baseline including sociodemographic and socioeconomic characteristics, health behaviours, a range of physical, mental and cognitive chronic health conditions and their consequences (disability and subjective global health ratings). The main limitations are the relatively small sample size, the loss to follow up, and the fact that not all chronic diseases were ascertained with equivalent rigour. Reassuringly, there were almost no statistically significant differences between those for whom vital status was and was not ascertained at follow-up, consistent with our impression that most failures to trace baseline participants were accounted for by the fairly random demolition of homes to make way for an IT superhighway.

The overall mortality rate of 70/1000 per year was slightly higher than national estimates of mortality for urban populations, accounted for entirely by an excess of male mortality in our cohort. The fact that 85% of deaths occurred at home, and 77% received no medical attention for their final illness underlines the inherent difficulty in estimating accurate mortality rates among older people using official registration procedures. The effect size for male gender in our study (HR 2.1, 95% CI 1.5-3.0 in the final model) was also much larger than that reported for those aged 65 and over in the Indian National Family Health Survey (OR 1.09, 95% CI 1.01-1.15) [9], and in a previous large verbal autopsy study in Chennai (M/F mortality ratio 1.28 for those aged 65-69, 1.15 age 70-74 and 0.80 for those aged 75 years and over) [45]. Our estimate is more consistent with those reported from developed countries, and may suggest a selective under-registration of deaths among older men in some previous Indian studies.

Our results show that male sex, mid upper arm circumference, age, disability and cognitive impairment/dementia are the parsimonious predictors of mortality among older people in Chennai, which, assuming causal relationships free of confounding account collectively for 90% of the observed mortality in the cohort under study. The impressive predictiveness of this model would seem to allow little scope for the salience to mortality of other unobserved or observed but misclassified factors. However,

1) it is likely that we underascertained diabetes, stroke and ischaemic heart disease at baseline because of reliance on self-reported clinical diagnoses, which would be biased because of low levels of help-seeking, low detection, and unclear communication of diagnoses to patients. We may therefore have underestimated the effect of some or all of these chronic physical health conditions upon mortality, some of which may have been captured instead in the sizeable independent effect of self-reported disability.

2) while age and sex cannot be modified it would be important, given the size of their effects, to understand better the mechanisms through which these are mediated. The effect of sex was not explained by any of the many compositional differences between men and women observed in our study, with the partial exception of lifelong and heavy problem drinking, which was more or less a male preserve, and which, upon adjustment, attenuated slightly the effect of male sex on mortality.

The absence of certain associations, consistently observed among younger people and in developed countries was striking. We failed to observe any effects of socioeconomic status, assessed in terms of income, assets and food insecurity, upon mortality. This finding is consistent with the absence of a socioeconomic gradient for mortality among older people in the Indian National Family Health Survey [9], in the study of older people in Wuhan, China [6], and with some research from developed countries [15]. It has been suggested that selective mortality in early life among those exposed to harsh economic deprivation, with only the fittest surviving to old age, may explain these findings [9]. The absence of any association with tobacco use is also consistent with Indian data suggesting an attenuation of effect in older people [46]. This finding may also be explained by selective mortality in earlier life. As noted in previous Indian research [10], and among older people in developed countries [17,18] undernutrition (highly prevalent in India), as opposed to obesity (still relatively rare) was by far the more potent risk factor for mortality in our cohort.

Some of the independent risk factors identified in our study may be amenable to preventive interventions. Undernutrition among older people accounted independently for 44% of deaths, and, in principle, is a modifiable exposure. More research is required to understand whether the excess mortality is accounted for by low calorie intake or specific micronutrient deficiency, or both. There are as yet no data from India on the prevalence of iron, B12 and folate deficiency among older people, but anemia is highly prevalent [47]. Undernutrition may be associated with underhydration that could be an important cause of mortality during hot and dry spells. In India, dementia is seen as a normal part of ageing [48], but it is also stigmatized [49], and associated with carer psychological and economic strain [50,51]. Community medical services fail to meet the needs of older people with dementia, because of their focus upon acute 'treatable' conditions, their lack of outreach, and their inability to provide education and long-term support to family carers [52]. A pilot randomized controlled trial of a home-based carer education and support intervention for people with dementia in Goa substantially reduced carer strain and was associated with a marginally non-significant 66% reduction in mortality in the intervention arm (OR 0.34, 95% CI 0.01-1.03) [53]. While dementia, among older people, is the major independent contributor to disability and dependency, comorbidity with other chronic physical and mental health conditions is the norm [54-56]. Thus, community-based interventions would be most efficiently incorporated into horizontal programs addressing the generic needs of frail, dependent older people and their carers, These could include attention to nutrition, hydration, immobility, behavioural disturbance, incontinence, sensory impairment, depression, hopelessness, and social isolation, whether arising from cognitive, mental or physical disorders, or a combination of these factors.

## Conclusions

The current priority agenda for preventing deaths from chronic diseases in LMIC [57] may be less relevant to older people, who account for nearly three quarters of chronic disease deaths in these regions. A more balanced approach to prevention of chronic disease deaths requires some attention to proximal risk factors in older people. Smoking and obesity seem much less relevant than in younger people. Undernutrition is preventable. Dementia can be conveniently be addressed, along with other, often comorbid chronic diseases, through interventions targeting frail dependent older people. The development and evaluation of such packages of care should be considered as an urgent priority, consistent with the Madrid International Plan for Action on Ageing call for primary and secondary healthcare and long-term care to meet the needs of older persons, eliminating social and economic inequalities.

## List of abbreviations

10/66 DRG: 10/66 Dementia Research Group; ADI: Alzheimer's Disease International; CDR: Clinical Dementia Rating; DSM-IV: Diagnostic and Statistical Manual of Mental Disorders, 4^th ^edition; ICD 10: International Classification of Diseases, 10th edition; IT: information technology; LMIC: low and middle income countries; MCI: Mild cognitive impairment; PARF: population attributable risk fraction; SMR: standardized mortality ratio; SD: standard deviation; Rs: Rupees (Indian); MV: Number of missing values; WHODAS 2.0: World Health Organization Disability assessment schedule, version 2.0

## Competing interests

The 10/66 Dementia Research Group works closely with Alzheimer's Disease International (ADI), the non-profit federation of 77 Alzheimer associations around the world. ADI is committed to strengthening Alzheimer associations worldwide, raising awareness regarding dementia and Alzheimer's Disease and advocating for more and better services for people with dementia and their caregivers. ADI is supported in part by grants from GlaxoSmithKline, Novartis, Lundbeck, Pfizer and Eisai

## Authors' contributions

The study was conceived and designed collectively by all of the authors. ATJ coordinated and conducted the field work for this study under the local supervision of JDW. ATJ and MJP conducted the analyses and wrote the first draft of this paper. All authors read and approved the final version.

## Pre-publication history

The pre-publication history for this paper can be accessed here:

http://www.biomedcentral.com/1471-2458/10/366/prepub
